# A bifunctional enzyme belonging to cytochrome P450 family involved in the *O*-dealkylation and *N*-dealkoxymethylation toward chloroacetanilide herbicides in *Rhodococcus* sp. B2

**DOI:** 10.1186/s12934-021-01544-z

**Published:** 2021-03-04

**Authors:** Hong-ming Liu, Meng Yuan, Ai-min Liu, Lei Ren, Guo-ping Zhu, Li-na Sun

**Affiliations:** 1 The Research Center of Life Omics and Health, Anhui Provincial Key Laboratory of the Conservation and Exploitation of Biological Resources, Anhui Normal University, Wuhu, 241000 Anhui People’s Republic of China; 2 Eco-Environmental Protection Research Institute, Shanghai Academy of Agricultural Sciences, Shanghai, 201403 People’s Republic of China; 3 College of Coastal Agricultural Sciences, Guangdong Ocean University, Zhanjiang, 524088 China

**Keywords:** Chloroacetanilide herbicides, *O*-Dealkylation, *N*-Dealkoxymethylation, *Rhodococcus*, P450 family oxygenase

## Abstract

**Background:**

The chloroacetamide herbicides pretilachlor is an emerging pollutant. Due to the large amount of use, its presence in the environment threatens human health. However, the molecular mechanism of pretilachlor degradation remains unknown.

**Results:**

Now, *Rhodococcus* sp. B2 was isolated from rice field and shown to degrade pretilachlor. The maximum pretilachlor degradation efficiency (86.1%) was observed at a culture time of 5 d, an initial substrate concentration 50 mg/L, pH 6.98, and 30.1 °C. One novel metabolite *N*-hydroxyethyl-2-chloro-*N*-(2, 6-diethyl-phenyl)-acetamide was identified by gas chromatography-mass spectrometry (GC–MS). Draft genome comparison demonstrated that a 32,147-bp DNA fragment, harboring gene cluster (*EthRABCD*_*B2*_), was absent from the mutant strain TB2 which could not degrade pretilachlor. The *Eth* gene cluster, encodes an AraC/XylS family transcriptional regulator (EthR_B2_), a ferredoxin reductase (EthA_B2_), a cytochrome P450 monooxygenase (EthB_B2_), a ferredoxin (EthC_B2_) and a 10-kDa protein of unknown function (EthD_B2_). Complementation with *EthABCD*_*B2*_ and *EthABD*_*B2*_*,* but not *EthABC*_*B2*_ in strain TB2 restored its ability to degrade chloroacetamide herbicides. Subsequently, codon optimization of *EthABCD*_*B2*_ was performed, after which the optimized components were separately expressed in *Escherichia coli*, and purified using Ni-affinity chromatography. A mixture of EthABCD_B2_ or EthABD_B2_ but not EthABC_B2_ catalyzed the *N-*dealkoxymethylation of alachlor, acetochlor, butachlor, and propisochlor and *O*-dealkylation of pretilachlor, revealing that EthD_B2_ acted as a ferredoxin in strain B2. EthABD_B2_ displayed maximal activity at 30 °C and pH 7.5.

**Conclusions:**

This is the first report of a P450 family oxygenase catalyzing the *O*-dealkylation and *N-*dealkoxymethylation of pretilachlor and propisochlor, respectively. And the results of the present study provide a microbial resource for the remediation of chloroacetamide herbicides-contaminated sites.
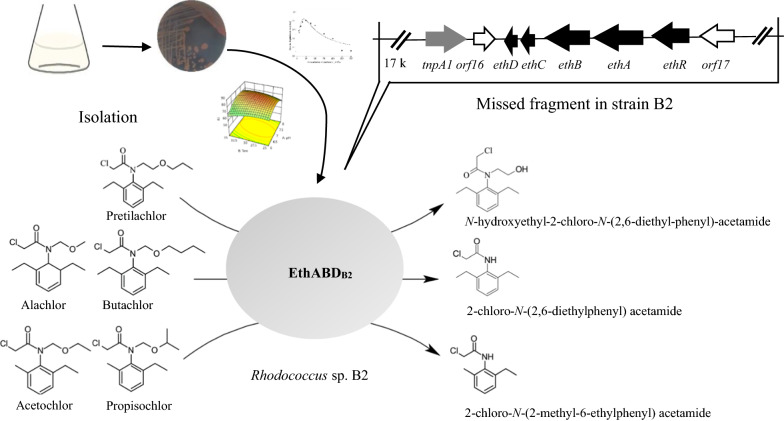

## Introduction

Chloroacetamide herbicides are preemergence herbicides utilized to control broadleaf weeds and annual grasses in the cultivation of soybeans, corn, rice and many other crops [1, 2]. The primary representative chloroacetamide herbicides, including acetochlor, alachlor, propisochlor, metolachlor, butachlor and pretilachlor are *N*-alkoxyalkyl-*N*-chloroacetyl-substituted aniline derivatives based on their structures. Due to their excessive application and chemical stability, chloroacetamide herbicides have been detected in the surface water, groundwater and drinking water in many countries [3–6]. Chloroacetamide herbicides have been reported to be highly deleterious to many aquatic organisms and are known to be carcinogenic to humans [7, 8].

Pretilachlor is a pre-planting or post-emergence herbicide used to eliminate broad-leaved weeds, grasses and sedges in rice fields [9]. Pretilachlor residue in soil can damage rice leaves and toxic to cyanobacteria [10, 11]. Pretilachlor exposure was shown to lead to apoptosis, immunotoxicity, endocrine disruption and oxidative stress in gestating zebrafish [12] and hepatic P4502B subfamily-dependent enzyme activity in rat liver [13]. Therefore, great concerns have been raised about elucidating the degradation mechanism of chloroacetamide herbicides.

Microbial metabolism is considered to be an important method for the removal of chloroacetanilide herbicides in ecosystems [14, 15]. At present, many chloroacetamide herbicides-degrading microorganisms have been reported, the initial metabolic steps of which primarily involves two pathways: glutathione mediation reaction [16, 17] and *N*-dealkylation [18, 19]. *N*-Dealkylation is primarily performed by enzymes in the Rieske non-heme iron oxygenase (RHO) and cytochrome P450 families in living organisms [20, 21]. Chen et al. reported an RHO system, CndABC, that can execute the *N*-dealkylation toward acetochlor, alachlor and butachlor, but not toward propisochlor, pretilachlor or metolachlor, in Sphingomonad strains DC-2 and DC-6 [18]. Wang et al. showed that *Escherichia coli* expressing *EthBAD*_*T3-1*_ (from *Rhodococcus* sp. T3-1) acquired *N*-deethoxymethylase activity toward acetochlor resulting in the conversion of acetochlor to CMEPA, whereas this activity was not observed toward metolachlor or pretilachlor [19, 22]. Currently, the molecular mechanism for the dealkylation of propisochlor, metolachlor and pretilachlor by microorganisms remains unknown.

The strain *Rhodococcus* sp. B2 was isolated from a rice field in which pretilachlor had been applied for many years. Strain B2 can degrade pretilachlor via an initial reaction of *O*-dealkylation. In the present study, the components of gene cluster *EthRABCD*_*B2*_ were cloned and their function were verified in *Rhodococcus* sp. TB2, Codon optimization of the *EthABCD*_*B2*_ gene cluster was performed, and the genes were individually expressed in *Escherichia coli*, and purified using Ni-affinity chromatography. A mixture of EthABCD_B2_ or EthABD_B2_ but not EthABC_B2_ displayed *N-*dealkoxymethylation activity toward alachlor, acetochlor, butachlor and propisochlor and *O*-dealkylation activity to pretilachlor, revealing a new mechanism for the initial degradation of chloroacetamide herbicide.

## Materials and methods

### Chemical reagents, medium, isolation and characterization of pretilachlor-degrading bacteria

Butachlor, alachlor, acetochlor, metolachlor, propisochlor, pretilachlor, 2-chloro-*N*-(2,6-diethyl phenyl) acetamide (CDEPA) and 2-Chloro-*N*-(2-methyl-6-ethylphenyl) acetamide (CMEPA) was purchased from Alfa-Aesar (Shanghai, China). Acetonitrile (HPLC grade) was from Sigma-Aldrich (Shanghai, China). Other reagents used in the present study were the AR grade. The composition of mineral salts medium (MSM), Luria–Bertani medium (LB), as well as the method of isolation, characterization and identification of pretilachlor degradation strain were previously described by Liu et al. [23, 24]. Briefly, 2 g of soil, collected from the rice field (Jiangsu, China), was added to 100 mL MSM containing 50 mg/L pretilachlor and shaken at 180 rpm, 30 °C. Then, after 5 days, 5 mL culture was transferred into fresh MSM and cultured under the same conditions. The concentration of pretilachlor was determined by HPLC, and a culture capable of degrading pretilachlor was selected to dilute and bacterial pure culture which can produce transparent circle in the plate containing 100 mg/L pretilachlor was picked.

Strain B2 was identified in line with Bergey's Manual of Determinative Bacteriology [25] and by 16S rRNA gene sequence analysis. The 16S rRNA gene sequence of strain B2 was aligned with sequence in EzTaxon-e server database (https://www.ezbiocloud.net/). A phylogenetic tree was constructed with MEGA (version 7.0) [26] using the neighbor-joining method.

### Optimization of pretilachlor-degrading conditions

The effects of cultivation conditions on pretilachlor degradation were assessed using the response surface methodology with a central composite design (CCD) procedure. Where the experiment was designed and performed with Design Expert (version 12.0.3; StatEase, Inc. Minneapolis, USA). Three factors, pH, temperature and inoculum size, were considered independent variables (Table 1), while the degradation rate of 50 mg/L of pretilachlor by strain B2 after 5 days served as the response variable. 20 runs with three replicates were performed. And an uninoculated culture served as a control. Then the experimental data were used in an empirical model analysis (quadratic polynomial equation):

1 $$Y = B + \, \sum B_{i} X_{i} + \, \sum B_{ij} X_{i} X_{{\text{j}}} + \, \sum B_{ii} X_{i}^{2}$$

Y: predicted response; Xi and Xj: variables; B: constant; bi: linear coefficient; bij: interaction coefficient; bii: quadratic coefficient.

**Table 1:** The design table of central composite design (CCD)

Symbols	Factors	Levels of variables				
		− Alpha	Low (− 1)	Medium (0)	High (1)	+ Alpha
A	pH	5.3	6	7	8	8.7
B	Temperature (℃)	21.6	25	30	35	38.4
C	Inoculation (g/L)	0.03	0.1	0.2	0.3	0.37

### Kinetics of pretilachlor-degradation by strain B2

Batch experiments were performed to analyze the effects of the initial concentration of pretilachlor on the degradation efficiency. Degradation ability was detected at different pretilachlor concentrations (10, 20, 30, 50, 75, 100, 125, 150, 200, 250, 300 mg/L) under the optimum conditions. The pretilachlor degradation efficiency was detected by HPLC as described below. The data were fitted to the Andrews model of substrate inhibition kinetics [27]:

2 $$q = \frac{{q_{\max } S}}{{S + K_{S} + (S^{2} /K_{i} )}}$$

3 $$S_{m} = \sqrt {K_{S} \times K_{i} }$$

*S:* concentration of pretilachlor; *Ki*: inhibition constant; *Ks*: half- saturation constant; and *qmax*: maximum degradation efficiency of pretilachlor; *Sm*: the critical inhibitor concentration of pretilachlor.

### Plasmids, strains and culture conditions

The plasmids and strains used in the present study are shown in Additional file 1: Table S1. The primers are presented in Additional file 1: Table S2. *E. coli* was cultivated at 37 °C with antibiotics added if necessary. Strain B2 was grown in LB medium supplemented with 100 mg/L pretilachlor at 30 °C. The strain B2 derivatives and other strains were all cultivated at 30 °C in LB medium.

### Genome sequencing, annotation, and genome analysis

DNA extraction was in line with the method of Sambrook et al. [28]. Genome sequencing of strains B2 and TB2 was performed using the Illumina MiSeq system by GENEWIZ Co., Ltd. (Suzhou, China). The BLAST search against the databases of Nr protein, KEGG, COG and Swiss-Prot databases were used for annotation. To determine the absent DNA fragment in strain TB2, a genome comparison of strain B2 and TB2 was carried out using the Mauve1.2.3 [29]. Chromosome walking was performed with SEFA-PCR [30]. Protein sequences were aligned with the ClustalX program [31], after which MEGA 7.0 [26] was used to build phylogenetic tree.

### Functional complement in strains TB2 and R-XP

In order to avoid the codon usage bias in *Rhodococcus*, the original promoter of the *Eth* cluster from *Rhodococcus* sp.B2 was used to express in the following fragments. A 4964-bp fragment *EthRABCD*_*B2*_*,* a 4597-bp fragment *EthRABC*_*B2*_ and a 3785-bp fragment *EthABCD*_*B2*_, were cloned from the genome of strain B2. A 3388-bp fragment *EthABD*_*B2*_ was amplified from the plasmid pQEth3. The fragments *EthRABCD*_*B2*_ and *EthRABC*_*B2*_*,* were ligated into the *Hind*III*-Spe*I sites of the *Rhodococcus-Escherichia coli* shuttle vector pRESQ [32], yielding pQEth1 and pQEth2, respectively. The fragments *EthABCD*_*B2*_ and *EthABD*_*B2*_*,* were cloned into the shuttle vector pRESQ using a Gibson Seamless Assembly Kit (HaiGene Co., Ltd). The constructed vectors were first transformed to *E. coli* DH5α and then introduced into strains TB2 or R-XP by electrotransformation [33]. All the recombinant plasmids were confirmed by sequencing. The abilities of the recombinant plasmid-harboring *E. coli* DH5α and TB2 strains to degrade pretilachlor were detected by whole-cell biotransformation experiments according to the method of Liu et al. with some modifications [34]. Briefly, the post-log phase transformants were collected by centrifugation, washed with MSM two times, and then resuspended in 20 mL MSM to a final OD_600nm_ value of approximately 1.0. Each cell suspension was incubated with substrate at a final concentration of 100 mg/L and cultivated at 160 rpm and 30 °C. Samples were harvested at appropriate intervals, and the degradation metabolites were monitored by HPLC as described below.

### Expression of EthABCD and purification of the recombinant proteins

To express *EthABCD* and *EthABC* under T7 promoter, a 3234-bp and a 2867-bp fragment without original promoter were PCR amplified from strain B2 with the primer pairs pET-EthF1/pET-EthR, pET-EthF2/pET-EthR, respectively. The PCR products were cloned into plasmid pET-29a(+) to construct the recombinant plasmids pET-EthABCD and pET-EthABC. *E. coli* BL21(DE3) harboring pET-EthABCD or pET-EthABC was grown at 37 °C until OD_600_ value of 0.6, and then incubated at 16 °C for 12 h after adding 0.5 mM IPTG. Subsequently, the degradation function was verified by whole-cell transformation with the centrifugal collection cells according to the above description.

Fragments of the gene cluster *EthABCD*, synthesized by GenScript company depending on *E. coli* codon usage form, were amplified with the primers shown in Table 2. The products were ligated into the corresponding site of expression plasmid pET29a(+) and transformed as recombiant plasmids into *E. coli* BL21(DE3)pLysS. Each recombinant plasmid was verified by sequencing. And gene expression and recombinant proteins purification were performed as the description of Hussain et al. [35]. SDS-PAGE was used to determine the protein molecular weight, and the protein concentrations were calculated by the Bradford method [36].

**Table 2:** Analysis of variance (ANOVA) for the response surface quadratic model

Source	Sum of squares	Degrees of freedom	Mean square	F-value	p-value	
Model	1489.53	9	165.5	21.17	< 0.0001	Significant
A-pH	54.42	1	54.42	6.96	0.0248	
B-T	5.29	1	5.29	0.6762	0.4301	
C-I	89.97	1	89.97	11.51	0.0069	
AB	87.12	1	87.12	11.14	0.0075	
AC	45.51	1	45.51	5.82	0.0365	
BC	4.62	1	4.62	0.591	0.4598	
A^2^	1118.5	1	1118.5	143.06	< 0.0001	
B^2^	152.73	1	152.73	19.53	0.0013	
C^2^	8.27	1	8.27	1.06	0.3279	
Residual	78.19	10	7.82			
Lack of fit	60.88	5	12.18	3.52	0.0969	Not significant
Pure error	17.31	5	3.46			
Cor total	1567.72	19				

### Enzyme activity assays

The enzymatic degradation of several chloroacetanilide herbicides activity was assessed at 30 ℃ for 1 h in a 1 mL mixture comprising (20 mM Tris–HCl buffer pH 7.5, 0.15 μg EthB_B2_, 0.58 μg EthA_B2_, 0.17 μg EthC_B2_, 0.12 μg EthD_B2_, 1 mM NADH, 0.1 mM NaCl, 0.5 mM Fe^2+^, and 1 mM MgCl_2_ and 1 mM 2-mercaptoethanol). The reaction was started after adding the substrates at a final concentration of 0.5 mM, The assays were stopped by the addition of 2 mL of dichloromethane, and the disappearance of the substrates was monitored by HPLC. Metabolites were determined by GC–MS analysis as described below. One unit of enzymatic activity was defined as the amount of enzyme required to generate of 1 nmol of product per minute.

The optimal pH for the reaction mixtures at 30 °C was determined in four different buffers: 20 mM citrate buffer (pH 3.8–5.8); 20 mM Na_2_HPO_4_-citric acid (pH 6.0–8.0); 20 mM Tris–HCl buffer(pH 7.5–9); and 20 mM glycine–NaOH buffer (pH 8.5–10.0). The optimal reaction temperature was evaluated at the optimal conditions and different temperatures (10–70 °C). The effects of potential inhibitors or activators on the enzymatic activity were determined by adding various metal ions and chemical agents to the reaction systems and incubating the sample at 30 °C for 60 min. Enzyme activity in the absence of any additive compounds was defined as 100%.

### Chemical analysis

Samples were extracted as previously description [23]. HPLC (LC-20AD; Shimadzu, Japan) analyses were performed with a Kromasil 100-5C18 column (250 mm × 4.6 mm × 5 μm) using an injection volume of 20 μL. Acetonitrile/water (80:20, v/v, 0.8 mL/min) was used as the mobile phase, and the absorbance at 215 nm was measured with a SPD-20A wavelength absorbance detector. Metabolites were identified via GC–MS (Shimadzu QP2010 Plus) with a RTX-5MS column (15 m length × 0.25 mm id × 0.25 µm internal diameter) in split mode (1:20). The temperature program was as follows: 100 °C for 1.5 min, ramp at 50 °C/min to 260 °C (7 min hold), ramp at 50 °C/min to 300 °C (1 min hold). The detected mass range was from 70 *m/z* to 350 *m/z*. Helium (1.0 ml/min) was the carrier gas. For HPLC–MS/MS analysis, the samples were detected by an Triple-quadrupole LC–MS/MS system (Agilent 1260/6460, USA), which is equipped with an electrospray ionization (ESI) and operated in the positive polarity mode. Firstly, MS2-SCAN mode was used to detect the compounds in the peaks. Secondly, Product ion Scan mode was used to detect target compound with a scan range from 50 to 500 *m/z.* The mobile phase consisted of solvents A (ultrapure water, 20%) and B (acetonitrile, 80%) with a flow rate was 0.3 mL/min. The injection volume was 5 μL.

### Nucleotide sequence accession number

The GenBank accession numbers of the *Rhodococcus* sp. B2 16S rRNA gene, sequence of the gene cluster *EthRABCD*_*B2*_ and scaffold 51 are KM875453, KJ946935 and MW378985.

## Results

### Isolation, and identification of the pretilachlor-degrading strain B2

A pure bacterial culture with gram-positive and nonmotile cells was isolated and named B2. B2 Colonies were convex, opaque and red on LB agar after two days of cultivation. In addition, strain B2 tested positive for urease and catalase but negative for nitrate reduction, oxidase and starch hydrolysis. The 16S rRNA gene sequence showed that strain B2 had 100% similarity with strain *R. erythropolis* DSM43066^T^ and 99.93% similarity with *R. erythropolis* NBRC100887^T^, forming a subclade with these two strains (Additional file 1: Fig. S1). Therefore, strain B2 was preliminarily identified as *Rhodococcus* sp. basing on its characteristics.

### Optimization of the cultivation conditions for pretilachlor-degradation

Three factors (pH, temperature, and inoculum size) with significant effects on microbial degradation were selected as the cultivation conditions to optimize using the CCD model. The data in Additional file 1: Table S3 were used in multiple regression analysis, and response variable Y can be obtained using the following quadratic polynomial model equation:

4 $${\text{Y}} = {84}.{31} + {2}.00{\text{A}} - 0.{\text{622B}} + {2}.{\text{57C}} + {3}.{\text{3AB}} - {2}.{\text{39AC}} + 0.{\text{76BC}} - {8}.{\text{81A}}^{2} - {3}.{\text{26B}}^{2} - 0.{\text{76C}}^{2}$$

**Fig. 1:**
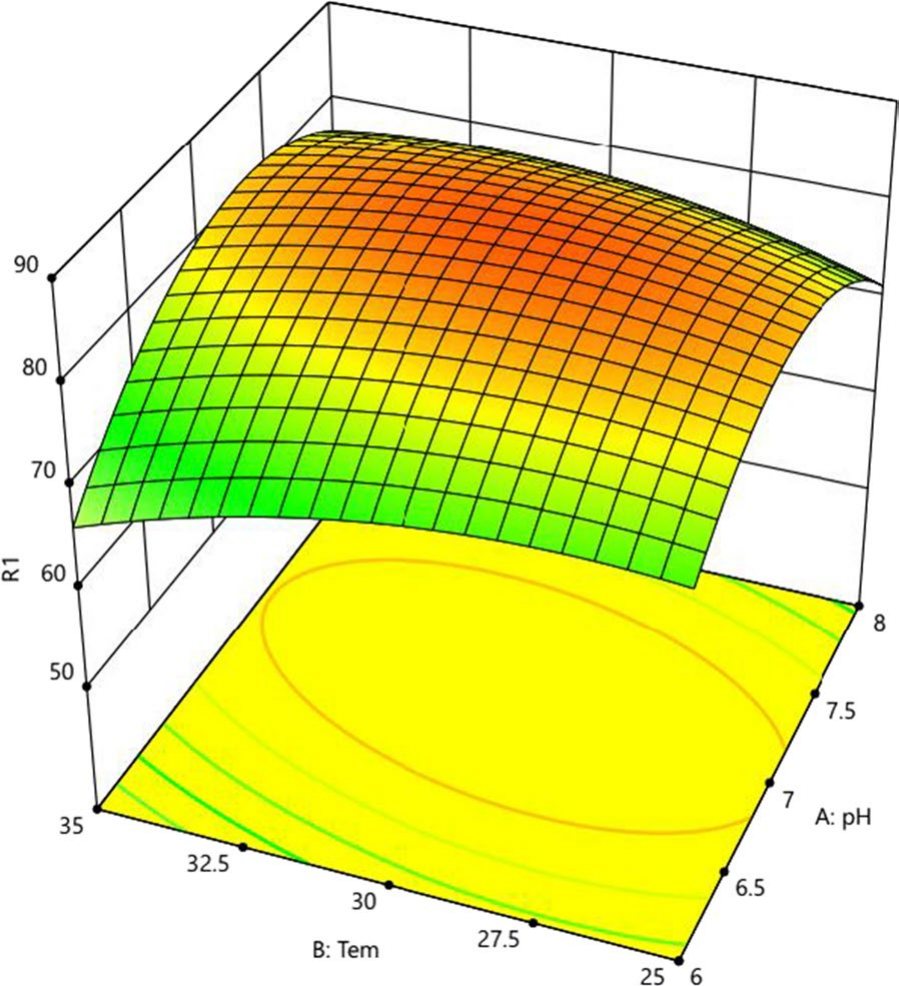
Response surface plot for the interaction effect of pH and temperature on the biodegradation activity of strain B2

The ANOVA results for the quadratic response surface model are shown in Table 2. The regression model for pretilachlor degradation was statistically significant (P < 0.05) with R^2^ = 0.9501, and the results showed that A, C, AB, AC, A^2^, B^2^ significantly affected the pretilachlor degradation by strain B2. Thus, pH, inoculum size had significant effects on the degradation rate. Based on the P values of AB and AC (0.0075 and 0.037), the pH-temperature and inoculum size-pH interaction effects on the pretilachlor degradation were highly significant. Therefore, a response surface analysis was conducted to determine the impacts of the interaction between pH and temperature on pretilachlor-degradation by strain B2 (Fig. 1). These results revealed the maximum rate of pretilachlor degradation by strain B2 was 86.1% under the optimal conditions of pH 6.98, 30.1 °C, and inoculum size of 0.3 g/L.

### Kinetics of pretilachlor degradation by strain B2

**Fig. 2:**
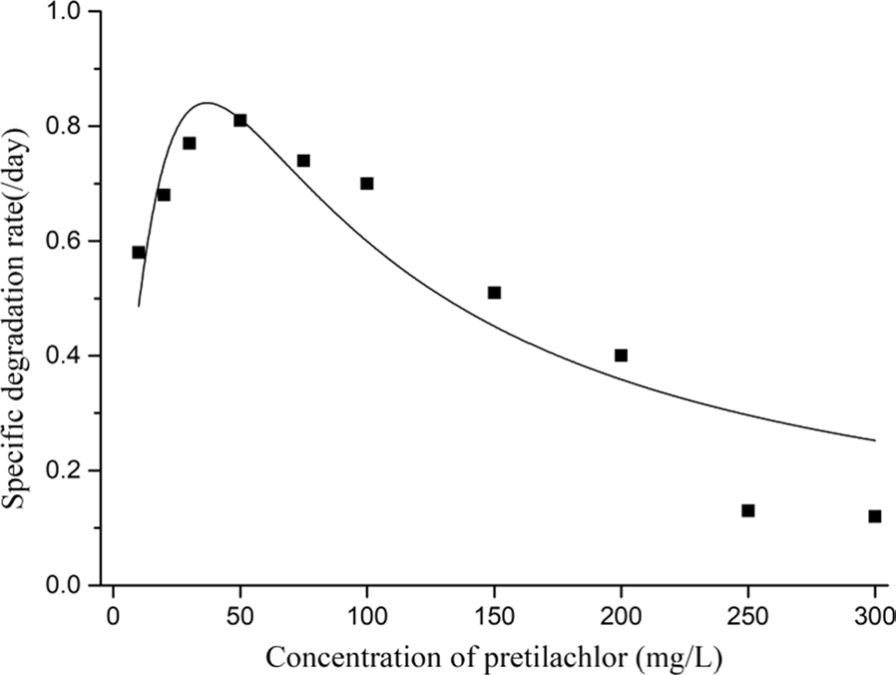
Relationship between initial pretilachlor concentration and specific degradation rate by strain B2

The effect of the initial pretilachlor concentration on the degradation of pretilachlor was calculated via nonlinear least squares regression analysis using Origin 9.0pro and the results are shown in Fig. 2. The kinetic parameters were as follows: *q*_*max*_ = 3.28 days^−1^, *K*_*S*_ = 53.51 mg/L, and *K*_*i*_ = 25.38 mg/L. The *S*_*m*_ was 36.84 mg/L, indicating that the theoretical efficiency of pretilachlor degradation was highest at this concentration, when the concentration of pretilachlor was more than 36.84 mg/L, the inhibition of strain B2 by the pretilachlor was obvious. This result may be attributed to the toxicity of pretilachlor to the assayed strain. The Andrews model was as follows:

$$q = \frac{3.28S}{{S + 53.51 + S^{2} /25.38}}$$

Statistical regression results revealed the parameters of pretilachlor degradation kinetics (Additional file 1: Table S4). The resulting correlation coefficient R^2^ = 0.8285, indicates that the model was an excellent fit to the experimental data. The ability of strain B2 to degrade pretilachlor degradation increased at low pretilachlor concentrations, but decreases at higher pretilachlor concentrations. And this kinetic model is helpful for predicting the microbial bioremediation of pretilachlor by strain B2.

### Identification of the metabolites resulting from pretilachlor degradation by strain B2

**Fig. 3:**
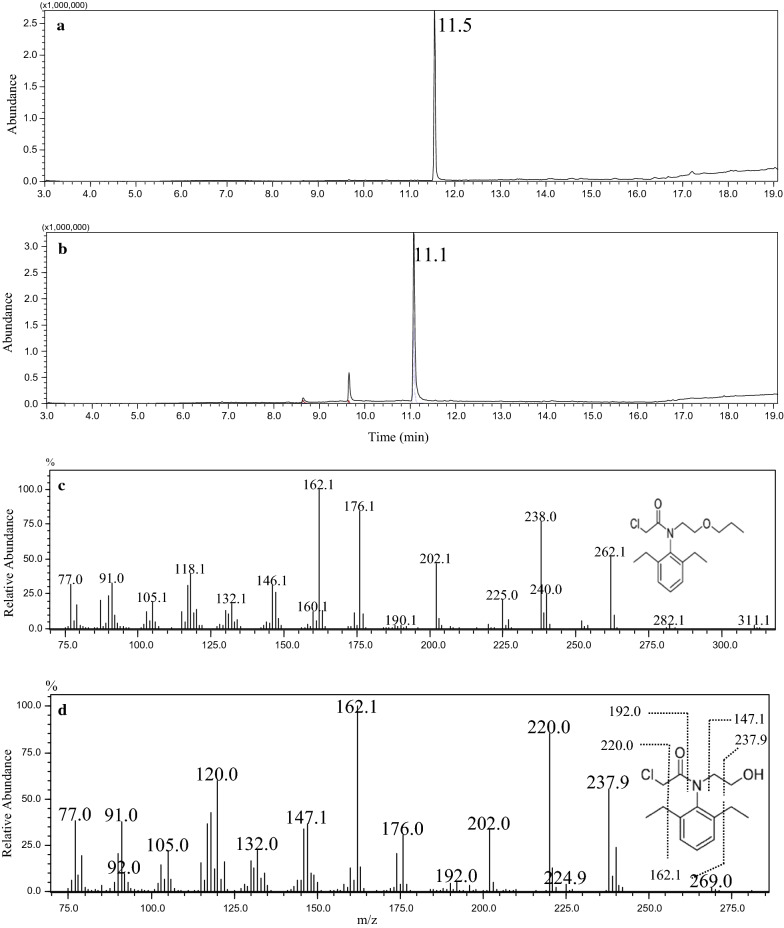
GC–MS profile of the metabolite produced during pretilachlor degradation by strain B2. **a**, **b** the GC spectra of the pretilachlor extract obtained from control sample and whole cell transformation of strain B2, respectively; **c** mass spectra for the peak at 11.5 min. **d** Mass spectra for the peak at 11.1 min

The products of pretilachlor degradation by strain B2 were detected by GC–MS. A compound from the control sample had the same RT as the pretilachlor standard (RT = 11.5 min, Fig. 3a). The molecular ion (M^+^) of peak (RT = 11.5 min) was 311 *m/z* with characteristic fragment ions mass spectral data showing a 96% match with pretilachlor in the NIST library (Fig. 3c).

In addition, a new metabolite appeared at a retention time of 11.1 min (Fig. 3b). As we could not obtain the standard for the metabolite, and it was identified using GC–MS analyses. The M^+^ peak of this product was 269 *m/z*, and the characteristic fragment ions were 237.9 *m/z* (M^+^ − CH_2_OH), 220.0 *m/z* (M^+^ − CH_2_Cl), 202.0 *m/z* (M^+^ − CH_3_ClOH), 192.0 *m/z* (M^+^ − CH_2_COCl), 176.0 *m/z* (M^+^ − (CH_2_CH_3_)_2_Cl), 162.1 *m/z* (M^+^ − (CH_2_CH_3_)_2_CH_2_Cl), 147.1 *m/z* (M^+^ − CH_2_COCl(CH_2_)_2_OH) and 132.0 *m/z* (M^+^ − CH_2_COCl(CH_2_)_2_OHCH_3_), (Fig. 3d), These mass spectral data of the metabolite correspond to *N*-hydroxyethyl-2-chloro-*N*-(2,6-diethyl-phenyl)-acetamide which has not previously been reported during pretilachlor degradation, indicating it to be a novel product. In addition, LC–MS/MS analysis demonstrated the presence of pretilachlor and the metabolite *N*-hydroxyethyl-2-chloro-*N*-(2,6-diethyl-phenyl)-acetamide, results that are consistent with the corresponding GC–MS analysis (Additional file 1: Figure S9). However, the metabolite could not be further metabolized by strain B2. Therefore, pretilachlor degradation process by strain B2 involves *O*-dealkylation, representing a new mechanism of initial chloroacetamide herbicide degradation.

### Screening of a mutant, TB2, defective in pretilachlor degradation

When grown on LB agar containing 100 mg/L pretilachlor, the colonies of strain B2 could produce a visible transparent halo, and *N*-hydroxyethyl-2-chloro-*N*-(2,6-diethyl-phenyl)-acetamide, which is more water-soluble than pretilachlor, was formed. In our present study, we observed that a few cells of strain B2 did not generate a transparent halo after continuous streaking on fresh LB agar plates, and one such isolate was named TB2. Resting cell transformation experiments revealed that TB2 could not metabolize pretilachlor (Additional file 1: Fig. S2), suggesting that the related gene responsible for *O*-dealkylation in pretilachlor degradation was deleted in the mutant TB2.

### Genome comparison between strains B2 and TB2

**Fig. 4:**
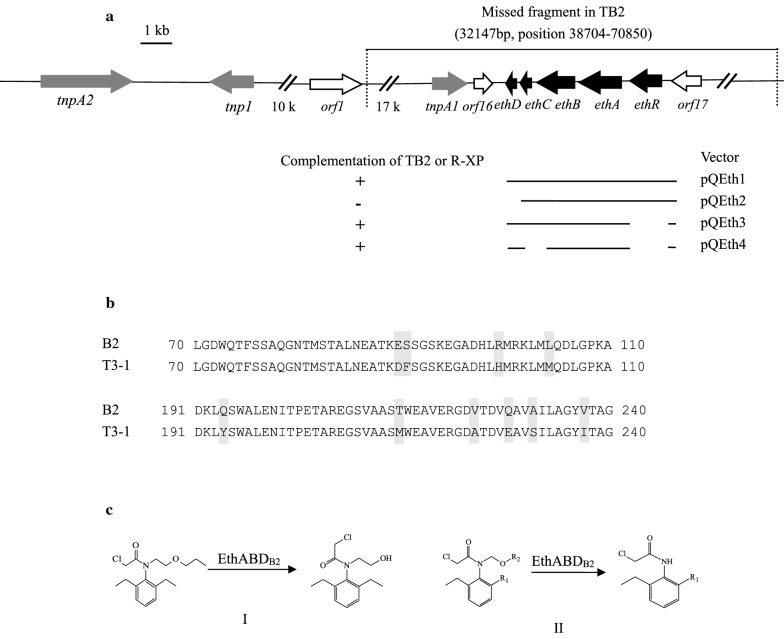
Physical map of the putative transposable element containing *EthRABCD*_B2_ gene cluster in *Rhodococcus* sp. B2 and degradation pathway of chloroacetanilide herbicides. **a** Arrows indicate the sizes, locations, and directions of transcription of the ORFs. Complementation of the *EthRABCD*_B2_-disrupted mutants with different regions is illustrated below the physical map. **b** The difference of the amino acid sequences of EthB from strain B2 and T3-1. Highlighted characters represent the different residues. **c** Proposed degradation pathway of pretilachlor, alachlor, acetochlor, propisochlor and butachlor by EthABD_B2_. I, pretilachlor. II, Alachlor, R1 = CH_3_CH_2_-, R2 = CH_3_-; Acetochlor, R1 = CH_3_-, R2 = CH_3_CH_2_-; Butachlor, R1 = CH_3_CH_2_-, R2 = CH_3_CH_2_CH_2_CH_2_-: Propisochlor, R1 = CH_3_-,R2 = (CH_3_)_2_CH-.Strain B2 cannot further metabolize three metabolites

The draft genomes of strains B2 and TB2 were sequenced with the Illumina MiSeq system and were shown to be 6,873,325 bp and 6,728,834 bp in length, respectively. Furthermore, a comparison of the genomes of strain B2 and TB2, resulted in the identification of a fragment from scaffold 51 of strain B2 that was absent in the genome of mutant TB2. The absence of this fragment was then verified by PCR. After which the flanking regions of scaffold 51 were confirmed by SEFA-PCR. Finally, a 115,851-bp fragment was acquired. And sequence comparison combined with PCR demonstrated that a 32,147-bp region of this fragment was absent in mutant TB2 (Fig. 4a).

### ORF analysis of the fragment absent in strain TB2

**Fig. 5:**
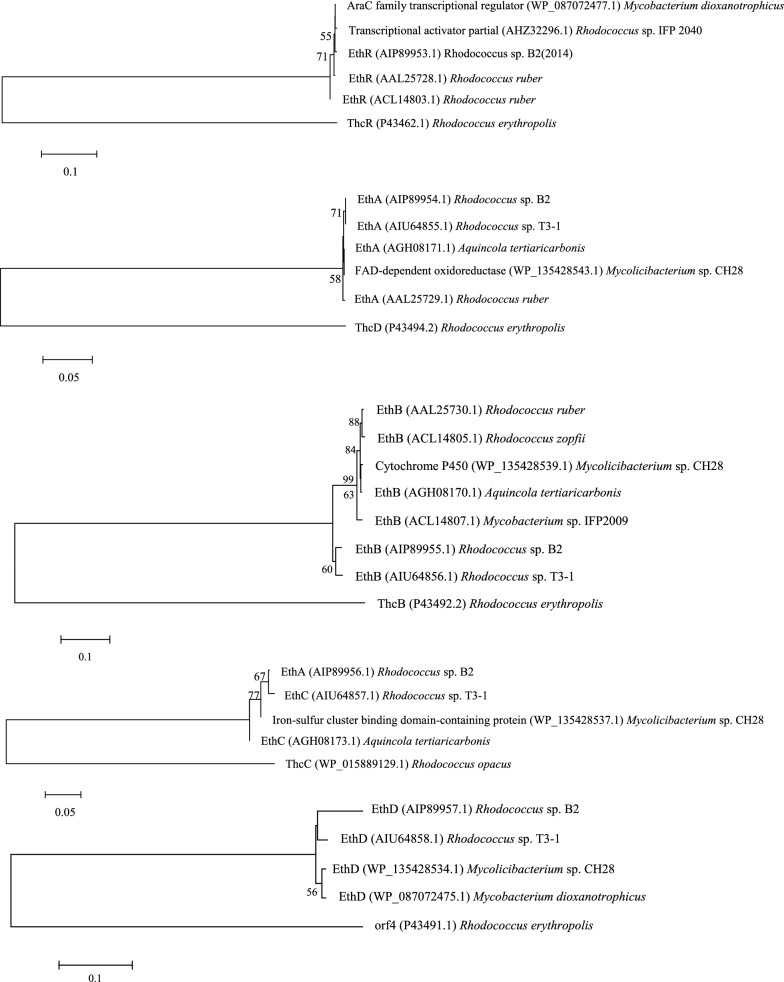
Phylogenetic tree of EthRABCD_B2_ and related proteins constructed by the neighbor-joining method. The branches corresponding to partitions reproduced in less than 50% of bootstrap replicates are collapsed. Name of the strains, proteins and their GenBank numbers are displayed in the phylogenetic tree. *Thc* gene cluster was used as an out-group The scale bar indicates amino acid residue substitutions per amino acid

A gene cluster consisting of five genes, *EthR*_*B2*_*, EthA*_*B2*_*, EthB*_*B2*_*, EthC*_*B2*_* and EthD*_*B2*_*,* was identified by an analysis of all ORFs, and the encoded amino acid sequence of the genes in the missing fragment were identified in NCBI (Additional file 1: Table S6). *EthR*_*B2*_, encoding the AraC/XylS family regulator (37 kDa), shares the highest similarity with the EthR from *Rhodococcus sp.* T3-1 (100%). *EthA*_*B2*_, encoding a ferredoxin reductase (43 kDa), shares the highest similarity with EthA from *Rhodococcus sp.* T3-1 (100%). *EthB*_*B2*_, encoding a cytochrome P450 oxidase (44 kDa), shares the highest similarity with EthB from *Rhodococcus sp.* T3-1 (97.5%). *EthC*_*B2*_, encoding a 2Fe-2S ferredoxin (11 kDa), shares the highest similarity with EthC from *Mycobacterium* sp. CH28 (99.06%). EthD_*B2*_, encoding a protein of unknown function (10 kDa), shares the highest similarity with EthD from *Mycobacterium* sp. CH28 (90.29%) (Fig. 5). The gene *EthB* was termed *EthB*_*B2*_ (EthB from strain B2), and its inferred amino acid sequence was aligned to that of EthB_T3-1_ from *Rhodococcus* sp. strain T3-1, as shown in Fig. 4b. Ten amino acid differences were observed between the two proteins, which may confer different physical properties to EthB_B2_. The high similarity of the two proteins indicated the occurrence of horizontal gene transfer event (Fig. 4a). The upstream *eth* gene cluster contains two gene fragments (*tnpA1,* and *tnpA2*) encoding the proteins displaying > 99% sequence identity with the Tn3 family transposase (TnpA) and one fragment (*tnp1*) belonging to the IS30 family transposase (Additional file 1: Table S6)*.* However, a transposase was not identified downstream of the *eth* gene cluster. In contrast, two transposons, IS3-type class II, flanked the *EthRABCD* gene cluster of *R. ruber* IFP 2001 [37].

### Functionally complementation of the *Eth* gene cluster in strain TB2

To determine the function of the gene cluster *EthRABCD*_*B2*_*,* the recombinant plasmid pQeth1 containing *EthRABCD*_*B2*_ was introduced into strain *E. coli* DH5α and strain TB2. The recombinant strain TB2 (pQeth1) acquired the ability to degrade pretilachlor and generate a visible transparent halo in LB agar supplemented with 100 mg/L pretilachlor, which was similar to that observed for strain B2. HPLC results showed that TB2 (pQeth1) could degrade pretilachlor, and exhibited the *O*-dealkylation activity (Additional file 1: Fig. S2). A similar phenomenon was found in strain *Rhodococcus* sp. R-XP(pQeth1). However, *E. coli* DH5α(pQeth1 or pQeth2) and strain TB2(pQeth2) failed to degrade pretilachlor, indicating that *EthRABCD*_*B2*_ was expressed at a low level or the original promoter could not promote transcription of the cluster in *E. coli*, and EthD_B2_ was essential for degradation. In order to verify the above hypothesis, the *EthABCD*_B2_ and *EthABC*_B2_ fragments under the control of the T7 promoter in vector pET-29a(+) were introduced into *E. coli* BL21(DE3). Whole-cell transformation assay results showed that the IPTG-induced suspension of *E. coli* BL21(DE3) harboring *EthABCD* (but not *EthABC*) was able to degrade pretilachlor, although with low activity (data not shown), These results indicated that *EthR*_*B2*_, *EthC*_*B2*_ gene are not essential and that the original promoter is important for the *Eth* gene cluster. Therefore, *EthABCD*_*B2*_ and *EthABD*_*B2*_ were reconstituted with original promoters to analyze the degradation of chloroacetanilide herbicides. The HPLC results showed that strain TB2 (pQeth3, and pQeth4) could convert pretilachlor, butachlor, alachlor, acetochlor and propisochlor to the corresponding metabolites, indicating that *EthD*_*B2*_ was likely a ferredoxin gene.

### Expression of the gene cluster *EthABCD*_*B2*_ and reconstitution of the chloroacetanilide herbicide degradation enzyme in vitro

The components *EthA*_*B2*_*, EthB*_*B2*_*, EthC*_*B2*_* and EthD*_*B2*_ were expressed in *E. coli* BL21(DE3)pLysS separately, and each recombinant protein was purified using Ni-affinity chromatography. The *Mw* value of the four proteins were consistent with the theoretically calculated values (Additional file 1: Fig. S3). Purified EthC_B2_-His6 and EthD_B2_-His6, were mixed with EthA_B2_-His6 and EthB_B2_-His6 in vitro. The results showed that the EthABD_B2_-His6 and EthABCD_*B2*_-His6 (not EthABC_*B2*_-His6) mixture showed pretilachlor-degrading activity indicating that EthD_B2_ is a ferredoxin. The catalytic activity of EthABD_B2_ toward pretilachlor was 3.41 ± 0.4 μmol/min/mg. EthABD_B2_ performed *N*-dealkoxymethylation to acetochlor, alachlor, propisochlor and butachlor, *O*-dealkylation to pretilachlor, but it was unable to degrade metolachlor. The GC–MS results showed that strain EthABD_B2_ could convert butachlor, alachlor, acetochlor and propisochlor to the corresponding metabolites CDEPA (for butachlor and alachlor) or CMEPA (for propisochlor and acetochlor) (Additional file 1: Fig. S4–S7). These metabolites are generated from C–N bond cleavage by *N*-dealkoxymethylation, and based on the comparison of the chemical structures of chloroacetanilide herbicides, the number of C-atoms between the N and O in the side chain affects the degradation. According to these results, the mechanism of pretilachlor degradation (*O*-dealkylation) was different from that of the other four chloroacetanilide herbicides (*N*-dealkylation), and the degradation pathway of chloroacetanilide herbicides by EthABD_B2_ was proposed (Fig. 4c). The recombinant strain TB2 (pQeth4) could not degrade metolachlor, suggesting that steric hindrance blocks enzyme–substrate interactions. EthB_B2_, a cytochrome P450 monooxygenase of the multicomponent system, plays a key role in chloroacetanilide herbicide degradation, and EthABD_B2_ from strain B2 has a broader substrate spectrum than that of the corresponding enzymes from strain T3-1. Thus, EthABD_B2_ is a better enzyme for practical bioremediation of chloroacetanilide herbicides.

### Characteristics of EthABD_B2_

The effects of different environmental factors on the enzymatic activity of EthABD_B2_ were determined (Additional file 1: Fig. S8). The enzyme activity was assessed at 10–65 °C, and was shown to function optimally at 30 °C (Additional file 1: Fig. S8B). In addition, EthABD_B2_ showed the enzyme showed high activity at pH 7.0–8.5, with an optimum pH of 7.5 in Tris–HCl buffer (Additional file 1: Fig. S8A), and decreased activity was lost observed at pH values below 4.0 or above 10.0. Metal ions play an important role in the enzyme activity. As shown in Additional file 1: Table S5, Fe^2+^ and Mg^2+^ could strongly enhance EthABD_B2_ activity, while Ca^2+^ could also slightly increase enzyme activity. However, the divalent cations Ba^2+^, Co^2+^ and Zn^2+^ slightly decreased enzyme activity, while Ag^+^, Cu^2+^, Hg^2+^, Ni^2+^ Cr^2+^ and Mn^2+^ significantly inhibited its enzyme activity. Chemical agents EDTA severely inhibited the enzyme activity, indicating that metal ions are required for its enzymatic reaction.

## Discussion

Glutathione S-transferase and cytochrome P450 play an important function in the detoxification of chloroacetanilide herbicides in plants and mammals [38, 39]. In microorganisms, CndABC, a monooxygenase system belonging to RHO family of *N*-dealkylase capable of catalyzing the *N*-dealkylation of butachlor, acetochlor and alachlor, was cloned from strains DC-2 and DC-6 [18]. *EthBAD*_*T3-1*_ from *Rhodococcus* sp. T3-1, expressed in *E. coli*, was previously shown to exhibit *N*-deethoxymethylase activity against acetochlor, but not pretilachlor and metolachlor [19, 22]. At present, we identified and characterized the function of the cytochrome P450 monooxygenase system EthABD_B2_ which was cloned from strain B2. EthABD_B2_, which exhibit a ten amino acids difference with the *EthBAD*_T3-1_ system, demonstrated the ability to catalyze the *O-*dealkylation or *N-*dealkoxymethylation of the chloroacetanilide herbicides pretilachlor, propisochlor, alachlor, acetochlor and butachlor. The different functions of the two proteins indicate that the key amino acid mutant broadens the substrate spectrum of this enzyme, indicting that the mutants of this protein could be attempted to degrade metolachlor in further research.

*EthRABCD* was first identified in the fuel oxygenate-degrading strain *R. ruber* IFP 2001 and exhibit *O*-dealkylation activity toward tert-amyl methyl ether (TAME), ethyl tert-butyl ether(ETBE) and methyl tert-butyl ether (MTBE) [37]. The *Eth* system, which is also present in gram-negative strain *Aquincola tertiaricarbonis* L108, with the exception of *EthR*, enables the efficient metabolization of MTBE, diethyl ether, TAME, ETBE, diisopropyl ether and tert-amyl ethyl ether(TAEE). However, this Eth monooxygenase system can not catalyze the degradtion of the synthetic ethers, including phenetole, and isopropoxybenzene, inferring that the nonreacting side chain link with the ether molecules may not exceed the size of the tert-amyl group [40]. In the present study, we enriched the function of Eth system in dealkylating O atom or dealkoxymethylating of N atom in synthetic compounds with larger residues, including pretilachlor, acetochlor, propisochlor, and butachlor.

The cytochrome P450 monooxygenase system EthRABCD_B2_ of *Rhodococcus* sp. B2 suffers from transposon-mediated recombination, resulting in *eth* loss mutants, e.g., *Rhodococcus* sp. TB2, which are unable to degrade pretilachlor. The deletion mechanism was shown to be similar to that observed in *R. ruber* IFP 2001 (IS3-type transposon element) but different from that from *A. tertiaricarbonis* L108 (rolling-circle IS91 type). The sequence similarity of the gene cluster *EthRABCD*_*B2*_ in the reported bacterial strains was > 90%, indicating that the *Eth* gene cluster *EthRABCD*_*B2*_ was horizontally transferred and that the transposons were likely the reason for its high mobility. Transposons in strains are rapidly adapted toward environmental transitions, such as substrate changes. This phenomenon is also true of enzymes required for the degradation of man-made xenobiotics, such as chloroacetanilide herbicides which have been applied globally for less than one hundred years [41]. The *Eth* gene cluster has been discovered in gram-positive strains, such as *R. ruber* IFP 2001 and *Rhodococcus* sp. T3-1 as well as gram-negative strains, such as *A. tertiaricarbonis* L108, suggesting that this gene cluster is highly conserved and has a complex transfer history.

EthABCD and EthABD, but not EthABC, showed activity against chloroacetanilide herbicides. A similar phenomenon was found in *Rhodococcus* sp. T3-1, indicating that EthD is a ferredoxin. EthC was predicted to be a ferredoxin, and we inferred that the EthABC system could potentially degrade other compounds. Interestingly, neither chloroacetanilide herbicides nor fuel oxygenates could be shown to serve as the natural inducers of the Eth system. The natural substrates should harbor *O*-alkyl or *N*-alkyl groups and be consistently present in the environment. The identification of this novel function of the *Eth* gene cluster can be exploited for the biodegradation of soil contaminated by gasoline ethers and chloroacetanilide herbicides.

## Conclusions

In the present study, a strain named *Rhodococcus* sp. B2 was isolated from a herbicide-contaminated field, and the optimum conditions (culture time, 5 days; initial substrate concentration, 50 mg/L; pH, 6.98; temperature, 30.1 °C) for efficient pretilachlor degradation (86.1%) were determined via response surface methodology. A novel product was detected during the pretilachlor biodegradation and identified. An DNA fragment absent from the mutant strain TB2 containing the functional gene cluster (called *EthRABCD*_*B2*_, first reported to be responsible for the *O*-dealkylation of the fuel oxygenate chemicals) was identified by genome comparison to strain B2. The recombinant enzyme EthABD_*B2*_ could degrade chloroacetanilide herbicides and catalyze the *N-*dealkoxymethylation of alachlor, acetochlor, butachlor and propisochlor and the *O*-dealkylation of pretilachlor. The broad substrate spectrum of EthABD_*B2*_ indicate the potential for bioremediation of environments contaminated by gasoline ethers and chloroacetanilide herbicides.

## Supplementary Information

**Additional file 1. Table S1** Strains and plasmids used in this study;** Table S2** PCR primers used in this study.** Table S3** Data of central composite design;** Table S4** The statistical regression results of pretilachlor degradation kinetics;** Table S5**. Effect of metal ions and EDTA on EthABD_B2_ enzyme activity;** Table S6** Deduced function of each ORF of scaffold 51 sequence containing the missing fragment;** Fig. S1** Phylogenetic tree constructed by the neighbour-joining method based on the 16S rRNA gene sequences;** Fig. S2** HPLC analysis of pretilachlor degradation by strain TB2 and TB2(pQEth1);** Fig. S3** SDS-PAGE(12%) analysis of the purified His6-tagged EthA, EthB, EthC and EthD;** Fig. S4** GC/MS analysis of the transformation of alachlor by the EthABD_B2_;** Fig. S5**. GC/MS analysis of the transformation of acetochlor by the EthABD_B2_;** Fig. S6** GC/MS analysis of the transformation of propisochlor by the EthABD_B2_;** Fig. S7** GC/MS analysis of the transformation of butachlor by EthABD_B2_;** Fig. S8** effects of pH (A) and temperature (B) on EthABD_B2_ activity;** Fig. S9** HPLC-MS/MS analysis of the pretilachlor and the intermediate metabolites catalyzed by the strain B2.

## Data Availability

All materials described within this manuscript, and engineered strains are available on request.
