# Supplementary material for: A bifunctional enzyme belonging to cytochrome P450 family involved in the O-dealkylation and N-dealkoxymethylation toward chloroacetanilide herbicides in Rhodococcus sp. B2
Source: Microb Cell Fact. 2021 Mar 4;20:61. doi: 10.1186/s12934-021-01544-z (PMC7934444; doi:10.1186/s12934-021-01544-z)
Supplement: Supplementary file 1 — Additional file 1. Table S1 Strains and plasmids used in this study; Table S2 PCR primers used in this study. Table S3 Data of central composite design; Table S4 The statistical regression results of pretilachlor degradation kinetics; Table S5. Effect of metal ions and EDTA on EthABDB2 enzyme activity; Table S6 Deduced function of each ORF of scaffold 51 sequence containing the missing fragment; Fig. S1 Phylogenetic tree constructed by the neighbour-joining method based on the 16S rRNA gene sequences; Fig. S2 HPLC analysis of pretilachlor degradation by strain TB2 and TB2(pQEth1); Fig. S3 SDS-PAGE(12%) analysis of the purified His6-tagged EthA, EthB, EthC and EthD; Fig. S4 GC/MS analysis of the transformation of alachlor by the EthABDB2; Fig. S5. GC/MS analysis of the transformation of acetochlor by the EthABDB2; Fig. S6 GC/MS analysis of the transformation of propisochlor by the EthABDB2; Fig. S7 GC/MS analysis of the transformation of butachlor by EthABDB2; Fig. S8 effects of pH (A) and temperature (B) on EthABDB2 activity; Fig. S9 HPLC-MS/MS analysis of the pretilachlor and the intermediate metabolites catalyzed by the strain B2. [file 12934_2021_1544_MOESM1_ESM.docx]

Supplementary information

**A** **Bifunctional Enzyme Belonging to Cytochrome P450 Family Involved in the** ***O*-Dealkylation and *N-*Dealkoxymethylation** **toward Chloroacetanilide Herbicides in** ***Rhodococcus* sp. B2**

Hong-ming Liu^a^, Meng Yuan^a^, Ai-min Liu^a^, Lei Ren^c^, Guo-ping Zhu^a^^*^, Li-na Sun^b*^

^a^ The Research Center of Life Omics and Health, Anhui Provincial Key Laboratory of the Conservation and Exploitation of Biological Resources, Anhui Normal University, Wuhu, 241000, Anhui, PR China

^b^ Eco-Environmental Protection Research Institute, Shanghai Academy of Agricultural Sciences, Shanghai, 201403, P.R. China

^c^ College of Coastal Agricultural Sciences, Guangdong Ocean University, Zhanjiang 524088, China

* Author for correspondence:

Guoping Zhu, E-mail: [gpz1996@yahoo.com](mailto:gpz1996@yahoo.com)

Lina Sun, E-mail: [slna@163.com](mailto:slna@163.com)

**Table S1. Strains and plasmids used in this study.**

| Strains or plasmids | Characteristics | Source or reference |
| --- | --- | --- |
| Strains |  |  |
| *Rhodococcus* sp. B2 | Degrades pretilachlor | This study |
| *Rhodococcus* sp. R-XP | unable to degrade pretilachlor | Our laboratory |
| *Rhodococcus* sp. TB2 | Mutant o f B2; unable to degrade pretilachlor | This study |
| *Escherichia coli* DH5α | F^−^ *recA1 endA*1 *thi-*1 *supE*44 *relA*1 *deoR* *Δ(lacZYA-argF)* U169 80d/lacZ ΔM15 | vazyme |
| *Escherichia coli* BL21(DE3) pLysS | F^-^, *ompT*, *hsdS_B_*(r_B_^-^,m_B_^-^),*dcm,gal*λ(DE3), pLysS, Cm^r^ | vazyme |
| Plasmids |  |  |
| pUC18-T | Cloning vector；Am^r^ | TaKaRa |
| pRESQ | *Rhodococcus-Escherichia coli* shuttle vector Km^r^ | Novagen |
| pQEth1 | pRESQ derivative containing *EthRABCD* under the control of the native promoter; Km^r^ | This study |
| pQEth2 | pRESQ derivative containing *EthRABC* under the control of the native promoter; Km^r^ | This study |
| pQEth3 | pRESQ derivative containing *EthABCD* under the control of the native promoter; Km^r^ | This study |
| pQEth4 | pRESQ derivative containing *EthABD* under the control of the native promoter; Km^r^ | This study |
| pET29a(+) | Expression vector; Km^r^ | vazyme |
| pET-EthABCD | pET-29a(+) derivative carrying EthABCD; Km^r^ | This study |
| pET-EthABC | pET-29a(+) derivative carrying EthABC; Km^r^ | This study |
| pETEthA | pET-29a(+) derivative carrying EthA; Km^r^ | This study |
| pETEthB | pET-29a(+) derivative carrying EthB; Km^r^ | This study |
| pETEthC | pET-29a(+) derivative carrying EthC; Km^r^ | This study |
| pETEthD | pET-29a(+) derivative carrying EthD; Km^r^ | This study |

**Table S2. PCR primers used in this study.**

| Primer | DNA Sequence (5´ to 3´)^a^ | Purpose |
| --- | --- | --- |
| EthF1 | GGACTAGTCTAGGTCCGGTCGACCTCATCCC | Forward primer to amplify *EthRABCD* with a Spe I site |
| EthF2 | GGACTAGTTCAGAACGCGTCGGGGACCTC | Forward primer to amplify *EthRABC* with a Spe I site |
| EthR1 | TCACCCAAGCTTGCGCTGGCAGCGCTTCAAGAACGCC | Reverse primer to amplify *EthRABCD* containing native promoter with a Hind III site |
| PF | CTCAAGCTATGCATCAAGCTTGCGCTGGCAGCGCTTCAAGAACGC | Forward primer to amplify native promoter with a Hind III site |
| PR | CGCCGATGATGATCATTCAGGCCTCCAAACCATCTGG | Reverse primer to amplify native promoter |
| EthF3 | GTTTGGAGGCCTGAATGATCATCATCGGCGCCGGGCAGG | Forward primer to amplify *EthABCD* |
| EthR3 | GCACACTGGCGGCCGTTACTAGTCTAGGTCCGGTCGACCTCATCCC | Reverse primer to amplify *EthABCD* with a SpeI site |
| PR2 | CCACGATCTGATACATTCACTTCGGGTAGATCCGCACGG | Reverse primer to amplify *EthAB* |
| EthF4 | GATCTACCCGAAGTGAATGTATCAGATCGTGGCCTGCTACGG | Forward primer to amplify *EthD* |
| EthR4 | GCACACTGGCGGCCGTTACTAGTCTAGGTCCGGTCGACCTCATCCC | Reverse primer to amplify *EthD* with a SpeI site |
| PET-EthF1 | TCACCCAAGCTTCTAGGTCCGGTCGACCTCATCCC | Forward primer to amplify *EthABCD* with a Hind III site |
| PET-EthR | GGGAATTCCATATGATCATCATCGGCGCCGGG | Reverse primer to amplify *EthABCD* with a NdeI site |
| PET-EthF2 | TCACCCAAGCTTTCAGAACGCGTCGGGGACCTC | Forward primer to amplify *EthABC* with a Hind III site |
| Pet-EthDF | GGGAATTCCATATGTACCAGATCGTTGCTTGCT | Forward primer to amplify *EthD* with a NdeI site |
| Pet-EthDR | CATGACCGCTCGAGGGTACGGTCAACTTCGTCACC | Reverse primer to amplify *EthD* with a XhoI site |
| Pet-EthCF | GGGAATTCCATATGCCGAAAATCACCTTCTCTCAGT | Forward primer to amplify *EthC* with a NdeI site |
| Pet-EthCR | CATGACCGCTCGAGGAACGCGTCGGGAACTTCGAGAC | Reverse primer to amplify *EthC* with a XhoI site |
| Pet-EthBF | GGGAATTCCATATGACCCTGTCTCTGGCTACCGCT | Forward primer to amplify *EthB* with a NdeI site |
| Pet-EthBR | ATGACCGCTCGAGTTTCGGGTAGATACGAACCGGCAGAG | Reverse primer to amplify *EthB* with a XhoI site |
| Pet-EthAF | GGGAATTCCATATGATCATCATCGGTGCTGGTCAGG | Forward primer to amplify EthA with a NdeI site |
| Pet-EthAR | CATGACCGCTCGAGACGAGCGGTAGCAACTTCACGAGC | Reverse primer to amplify EthA with a XhoI site |

| **Table S3 Data of central composite design** | | | | |  |  |
| --- | --- | --- | --- | --- | --- | --- |
| Run | A | B | C | Degradation rate of pretilachlor (%) | | |
| 1 | 0 | 0 | 0 | 82.24 | |  |
| 2 | -1 | 1 | -1 | 60.56 | |  |
| 3 | 1 | 1 | 1 | 78.41 | |  |
| 4 | -1 | 1 | 1 | 67.63 | |  |
| 5 | 0 | -1.68179 | 0 | 73.26 | |  |
| 6 | 0 | 0 | -1.68179 | 76.38 | |  |
| 7 | 0 | 0 | 0 | 82.64 | |  |
| 8 | 0 | 1.681793 | 0 | 76.08 | |  |
| 9 | 0 | 0 | 0 | 86.52 | |  |
| 10 | 1 | -1 | -1 | 75.63 | |  |
| 11 | -1 | -1 | -1 | 68.51 | |  |
| 12 | 1 | 1 | -1 | 73.92 | |  |
| 13 | 1 | -1 | 1 | 70.12 | |  |
| 14 | 0 | 0 | 1.681793 | 87.09 | |  |
| 15 | 0 | 0 | 0 | 83.26 | |  |
| 16 | 0 | 0 | 0 | 85.11 | |  |
| 17 | 1.681793 | 0 | 0 | 60.56 | |  |
| 18 | -1 | -1 | 1 | 79.5 | |  |
| 19 | 0 | 0 | 0 | 86.21 | |  |
| 20 | -1.68179 | 0 | 0 | 57.36 | |  |

**Table S4 The statistical regression results of pretilachlor degradation kinetics**

| Statistics | |
| --- | --- |
| Number of Points | 10 |
| Degrees of Freedom | 7 |
| Reduced Chi-Sqr | 0.011 |
| Residual Sum of Squares | 0.07701 |
| Adj. R-Square | 0.82852 |

| **Table S5.** Effect of metal ions and EDTA on EthABD_B2_ enzyme activity | |
| --- | --- |
| Substance | Relativeactivity (%) |
| None | 100 |
| Ba^2+^ | 78.2 ± 5.8 |
| Li^+^ | 91.28± 4.6 |
| Ag^+^ | 9.2 ± 5.4 |
| Fe^2+^ | 167.1 ± 6.8 |
| Mg^2+^ | 121.4 ± 7.2 |
| Co^2+^ | 89.4± 6.3 |
| Cr^2+^ | 21.4 ± 8.2 |
| Cu^2+^ | 31.9 ± 9.1 |
| Hg^2+^ | 1.2 ± 0.2 |
| Zn^2+^ | 71.2 ± 2.9 |
| Ca^2+^ | 101.7 ± 3.7 |
| Mn^2+^ | 32.3 ± 5.1 |
| Ni^+^ | 18.1 ± 6.3 |
| EDTA | 8.6 ± 1.4 |

**Table S6. Deduced function of each ORF of scaffold 51 sequence containing the missing fragment.**

| Gene name, proposed product(s) | Position in the bp fragment, product size (amino acids) | Homologous protein (GenBank accession no.) and source | % Identity |
| --- | --- | --- | --- |
| *orf1*, Reverse transcriptase | 709-2508, 599 | Group II intron reverse reverse transcriptase (WP_140189226.1), *Rhodococcus ruber* | 92.76 |
| *Orf2*, Methyltransferase | 3618-4361, 247 | Methyltransferase domain-containing protein (WP_017681401.1)  *Rhodococcus ruber* | 99.60 |
| *Orf3,* Hypothetical protein | 5409-5116, 97 | Hypothetical protein (WP_124259314.1) *Rhodococcus* | 98.97 |
| *Orf4*, Indolylacetylinositol arabinosyltransferase | 5887-7050, 387 | Indolylacetylinositol arabinosyltransferase (KXF84347.1) *Rhodococcus ruber* Chol-4 | 99.74 |
| *Orf5* Hypothetical protein | 7802-7137, 221 | Hypothetical protein (WP_017681398.1), *Rhodococcus ruber* | 99.55 |
| *Orf6*, Hypothetical protein | 7943-8251, 102 | Hypothetical protein (WP_006936885.1), *Rhodococcus* | 100 |
| *Orf7*, Lipid II flippase FtsW | 8446-9945, 499 | Lipid II flippase FtsW (CDZ90220.1), *Rhodococcus* ruber | 99.60 |
| *Orf8*, Hypothetical protein | 10696-10259, 145 | Hypothetical protein (WP_048774368.1), *Rhodococcus ruber* | 100 |
| *Orf9*, Hypothetical protein | 11214-10693, 173 | Hypothetical protein CQZ88_13450, (PND51554.1), *Rhodococcus* sp. ENV425 | 100 |
| *Orf10*, Integrase | 11228-12277, 349 | Tyrosine-type recombinase/integrase (WP_043797108.1), *Rhodococcus* | 100 |
| *Orf11*, Integrase | 12274-14097, 607 | Phage integrase family protein (WP_043797110.1), *Rhodococcus* | 99.84 |
| *orf12*, Aldehyde dehydrogenase | 14127-14549, 140 | Aldehyde dehydrogenase family protein (WP_045739656.1), *Actinoplanes rectilineatus* | 34.83 |
| *orf13*, Integrase | 16783-15650, 377 | Integrase family protein (ETT28371.1), *Rhodococcus rhodochrous* ATCC 21198 | 98.94 |
| *orf14*, Hypothetical protein | 17348-18331, 327 | Hypothetical protein (WP_048774373.1) *Rhodococcus ruber* | 99.38 |
| *Orf15*, Hypothetical protein | 19318-18965, 117 | hypothetical protein FF86_1001157 (KQM07901.1), *Frankia* sp. Cpl1-P | 41.33 |
| *TnpA*, Transposase TnpA | 19346-20440, 364 | Tn3 family transposase (WP_135422487.1), *Mycolicibacterium* sp. CH28 | 99.25 |
| *orf16*, Hypothetical protein | 20610-21275, 221 | Hypothetical protein (WP_135428527.1), *Mycolicibacterium sp.* CH28 | 100 |
| *EthD*, Unknown function protein | 21826-21530, 98 | EthD (WP_135428534.1), *Mycolicibacterium* sp. CH28 | 90.29 |
| *EthC*, Ferredoxin | 22226-21897, 109 | EthC (WP_135428537.1 ), *Mycolicibacterium* sp. CH28 | 99.06 |
| *EthB*, P450 oxygenase | 23425-22223, 400 | EthB (AIU64856.1), *Rhodococcus sp.* T3-1 | 97.5 |
| *EthA*, Ferredoxin reductases | 24763-23495, 422 | EthA (AIU64855.1), *Rhodococcus sp.* T3-1 | 100 |
| *EthR*, Regulator | 25912-24917, 331 | EthR ( AAL25728.1), *Rhodococcus ruber* | 99 |
| *Orf17*, Putative membrane protein | 27413-26325, 362 | Putative membrane protein (CDZ90219.1), *Rhodococcus ruber* | 99.71 |
| *Orf18*, ParA | 28772-28095, 225 | ParA family protein (WP_029540292.1), *Rhodococcus* | 100 |
| *Orf19*, cytochrome P450 | 30034-31287, 417 | Cytochrome P450 (WP_006931038.1), *Rhodococcus* | 100 |
| *orf20*, DUF5593 domain-containg protein | 31393-32376, 327 | DUF5593 domain-containg protein  (WP_160331281.1), *Rhodococcus ruber* | 100 |
| *orf21*, Hypothetical protein | 34207-32405, 600 | Hypothetical protein (WP_048774272.1), *Rhodococcus ruber* | 99.67 |

*Rhodococcus cerastii* C5^T^ F(FR714842)

*Rhodococcus yunnanensis* YIM 70056^T^ (AY602219 )

*Rhodococcus globerulus* DSM 4954^T^ (X80619)

*Rhodococcus jialingiae* djl-6-2^T^ (DQ185597)

*Rhodococcus qingshengii* djl-6^T^ (DQ090961 )

*Rhodococcus baikonurensis* GTC1041^T^ (AB071951)

***Rhodococcus* sp. B2**

*Rhodococcus erythropolis* NBRC 100887 (AP008957)

*Rhodococcus erythropolis* DSM 43066^T^ (X79289)

*Rhodococcus maanshanensis* M712^T^ (AF416566)

*Rhodococcus marinonascens* DSM 43752^T^ (X80617)

*Rhodococcus koreensis* DNP505^T^ (AF124343)

*Rhodococcus jostii* IFO 16295^T^ (AB046357)

*Rhodococcus wratislaviensis* NCIMB 13082^T^ (Z37138)

*Rhodococcus imtechensis* RKJ300^T^ (AY525785 )

*Rhodococcus percolatus* MBS1^T^ (X92114 )

*Rhodococcus opacus* DSM 43205^T^ (X80630)

*Rhodococcus nanhaiensis* SCSIO 10187^T^ (JN582175 )

*Rhodococcus triatomae* IMMIB RIV-085^T^ (AJ854055)

*Rhodococcus artemisiae* YIM 65754^T^ (GU367155)

99

93

67

96

53

72

80

78

93

57

59

97

68

95

96

98

0.005

**Fig. S1. Phylogenetic tree constructed by the neighbour-joining method based on the 16S rRNA gene sequences.** *Rhodococcus artemisiae* YIM 65754^T^ was used as the outgroup. Bootstrap values (numbers on branch nodes expressed as percentages of 1000 replications) greater than 50% are shown at branch points.

A
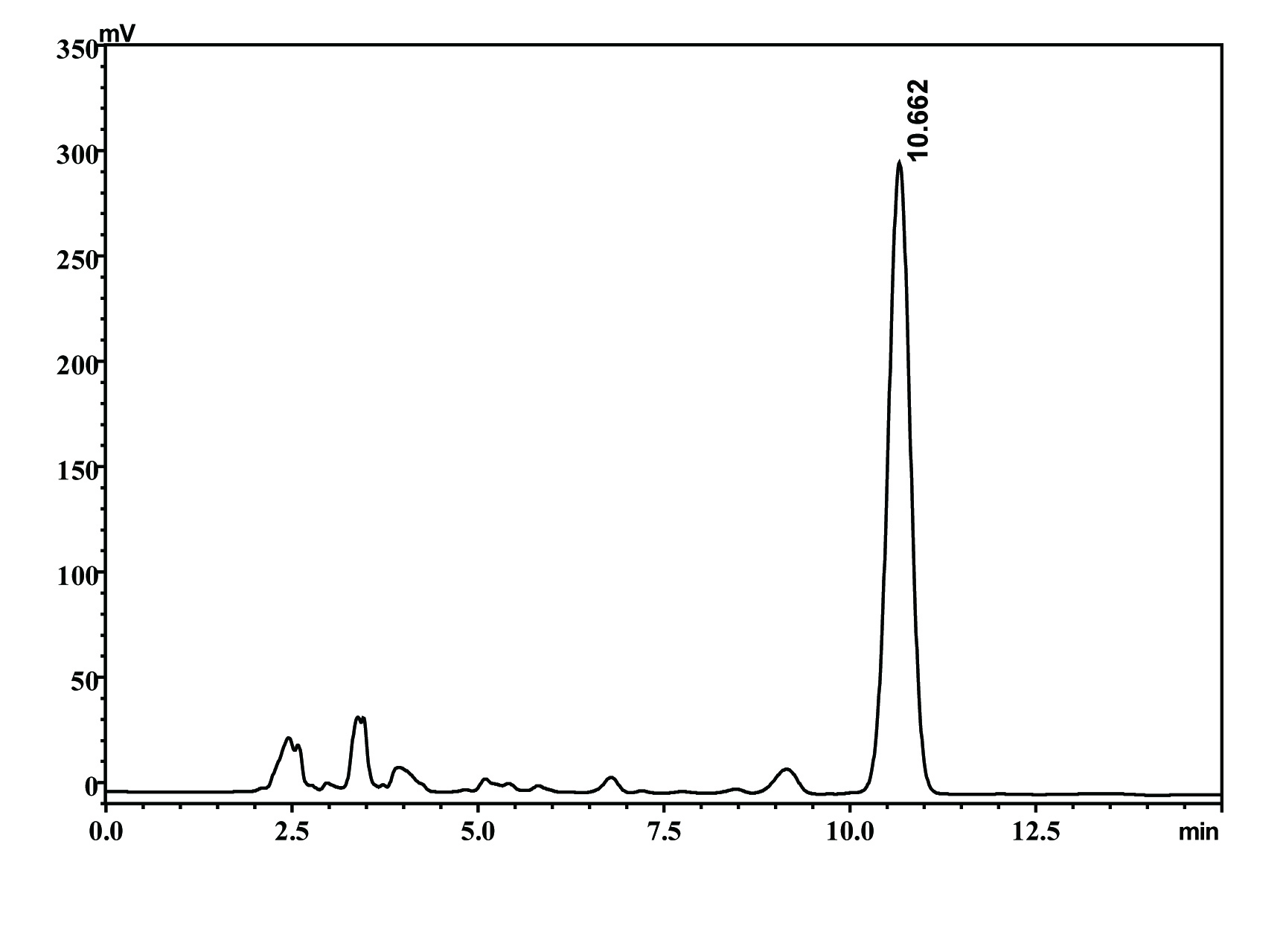


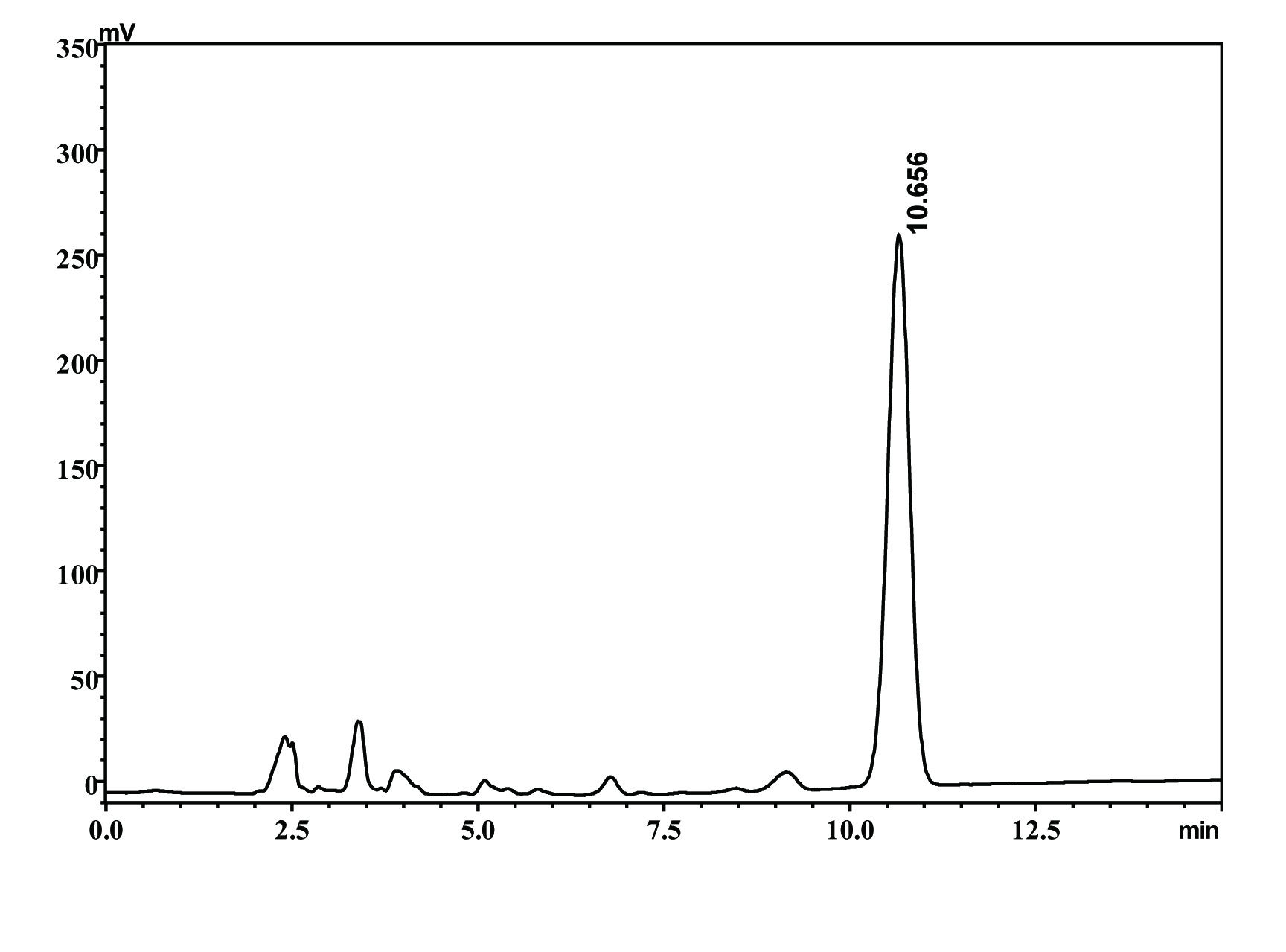
 B

C


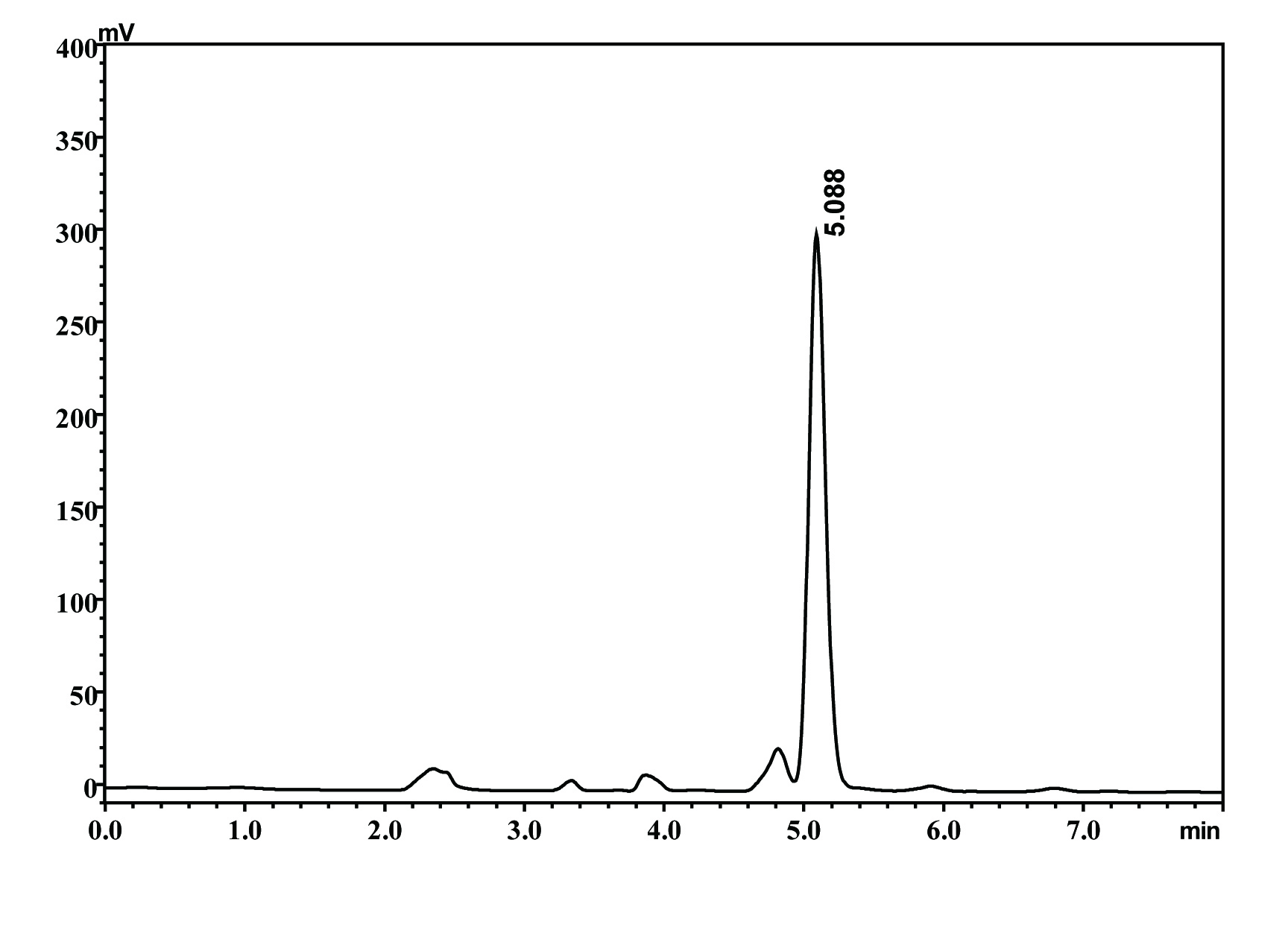


**Fig. S2. HPLC analysis of** **pretilachlor degradation by strain TB2 and TB2(pQEth1).** A, HPLC spectra of control sample; B and C, HPLC spectra of extract obtained from the culture at 48 h of TB2 and TB2(pQEth1), respectively; the medium used was MSM containing 100 mg/L pretilachlor; the peak at 10.656 min was pretilachlor.


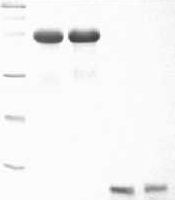


97.2

kDa M 1 2 3 4

66.4

44.3

29.0

20.1

14.3

**Fig. S3. SDS-PAGE(12%) analysis of the purified His6-tagged EthA, EthB, EthC and EthD.** Lane M, molecular weight markers. Lane 1, EthA; Lane 2, EthB; Lane 3, EthC; Lane 4, EthD.

A

8.6

9.8

Abundance

Time (min)

metabolite

alachlor

B

Time (min)

Abundance

standard

C


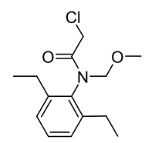


Relative Abundance

m/z

D


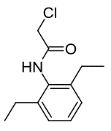


Relative Abundance

m/z

E


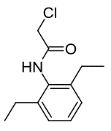


m/z

Relative Abundance

**Fig. S4. GC/MS analysis of the transformation of alachlor by the EthABD_B2_.** A, GC spectra of the extract obtained from alachlor transformation for enzyme EthABD_B2_; B, GC spectra of the standard; C and D, mass spectra for the peak with RT value of 9.8 min and 8.6 min. E, mass spectra for the standard.

A

Abundance

Time (min)

acetochlor

metabolite

8.2

9.7

B

standard

Time (min)

Abundance


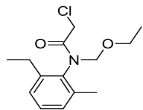
C

Relative Abundance

m/z

D


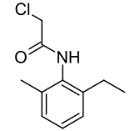


Relative Abundance

m/z

E


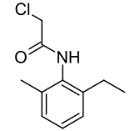


m/z

Relative Abundance

**Fig. S5. GC/MS analysis of the transformation of acetochlor by the EthABD_B2_.** A, GC spectra of the extract obtained from acetochlor transformation for enzyme EthABD_B2_; B, GC spectra of the standard; C and D, mass spectra for the peak with RT value of 9.7 min and 8.2 min; E, mass spectra for the standard.

A

Abundance

Time (min)

8.2

9.9

metabolite

propisochlor

B

m/z

standard

Time (min)

Abundance

C


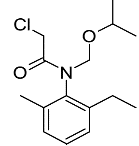


Relative Abundance

D


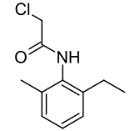


Relative Abundance

E


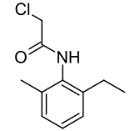


m/z

Relative Abundance

**Fig. S6. GC/MS analysis of the transformation of propisochlor by the EthABD_B2_.** A, GC spectra of the extract obtained from propisochlor transformation for enzyme EthABD_B2_; B, GC spectra of the standard; C and D, mass spectra for the peak with RT value of 9.9 min and 8.2 min; E, mass spectra for the standard.

A

B

Abundance

11.2

8.6

Time (min)

butachlor

metabolite

Abundance

Time (min)

standard

C


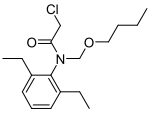


Relative Abundance

m/z


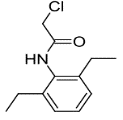
D

m/z

Relative Abundance

E


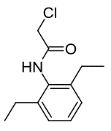


m/z

Relative Abundance

**Fig. S7. GC/MS analysis of the transformation of butachlor by EthABD_B2_.** A , GC spectra of the extract obtained from butachlor transformation for enzyme EthABD_B2_; B, GC spectra of the standard; C and D, mass spectra for the peak with RT value of 11.2 min and 8.6 min; E, mass spectra for the standard .


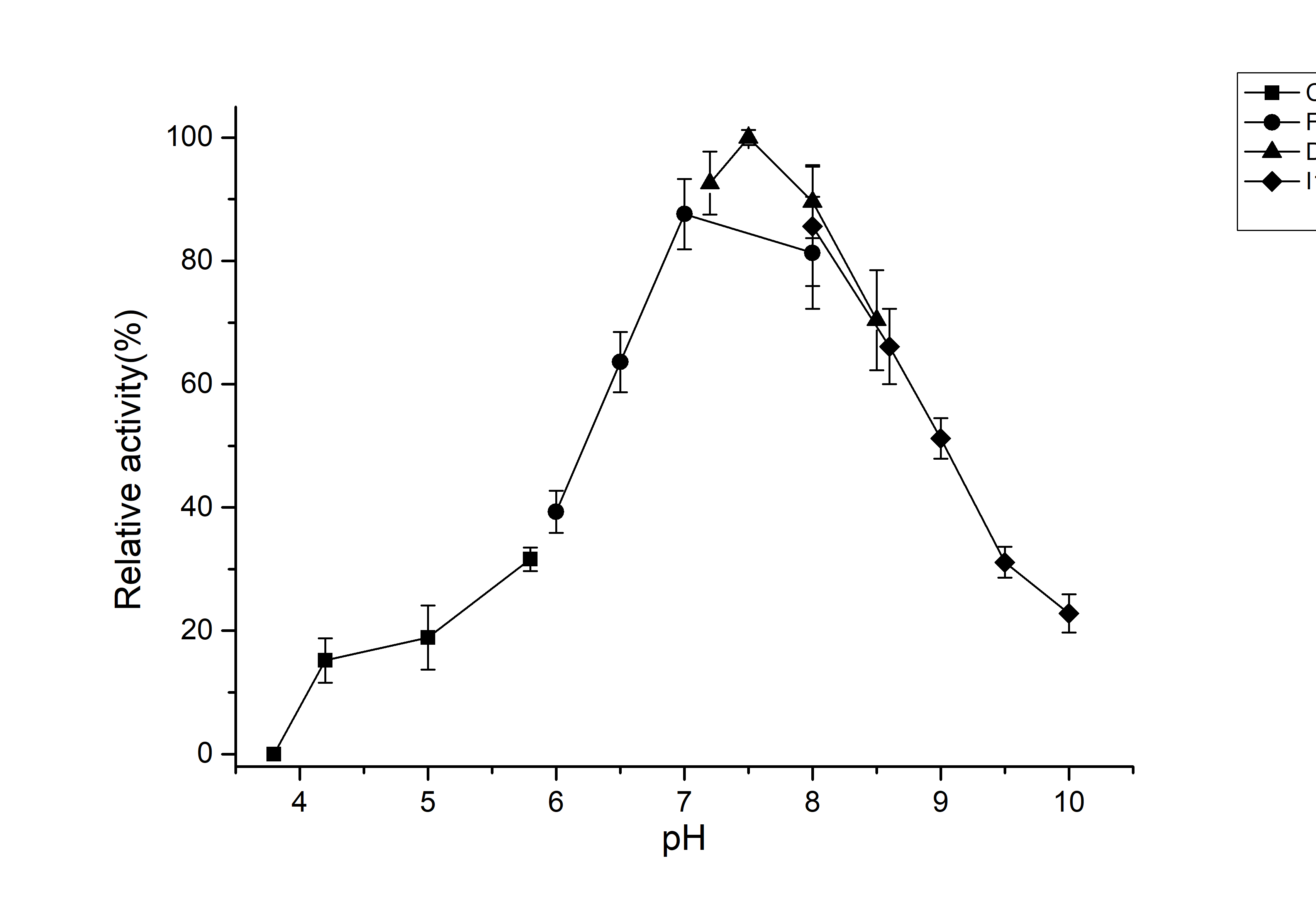
A

**
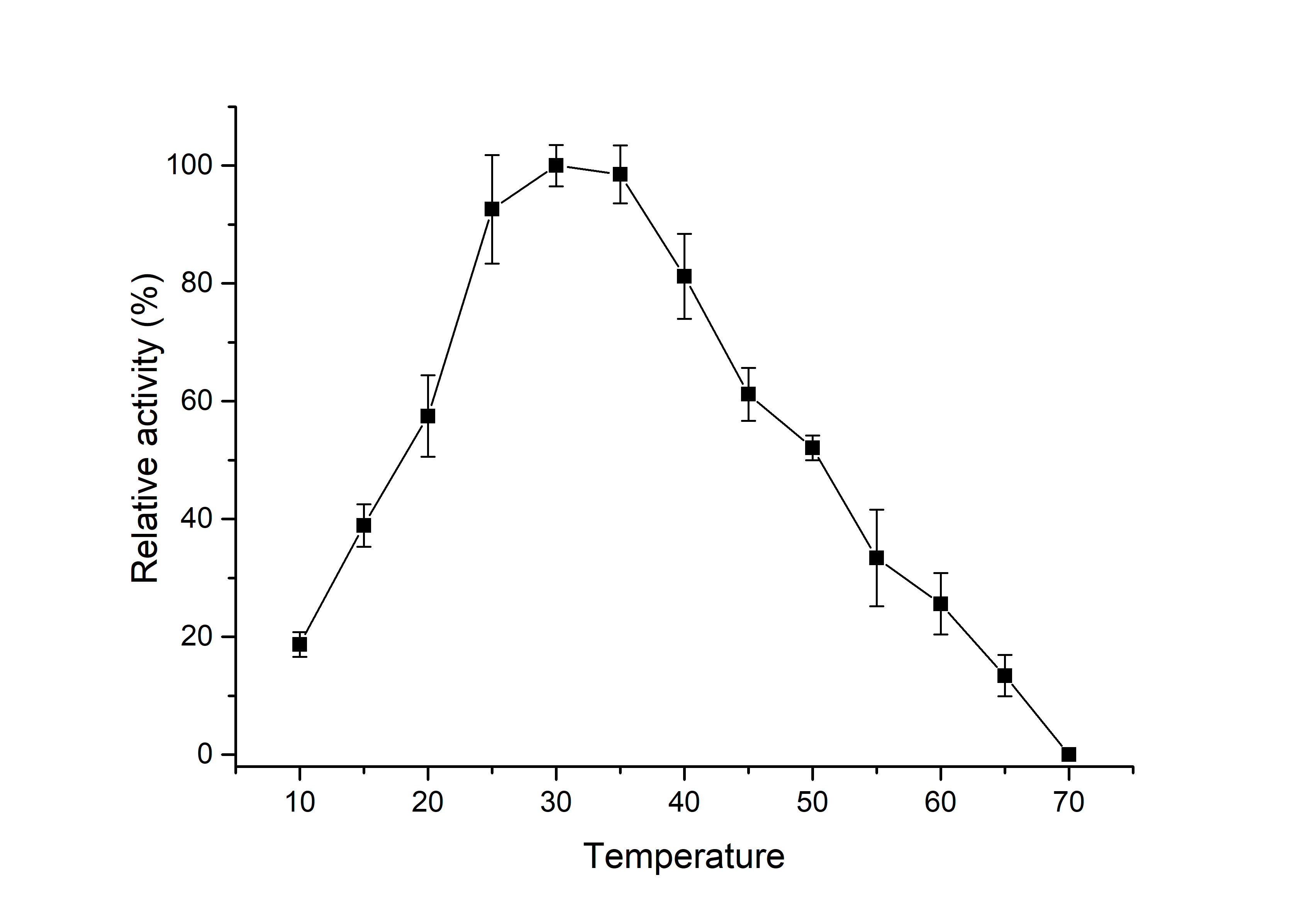
**

B

**Fig. S8** effects of pH (A) and temperature (B) on EthABD_B2_ activity

A


B

C

D

**C_14_H_19_ClNO**

**C_12_H_18_N**

**C_14_H_21_ClNO_2_**

**C_10_H_12_NO**

**C_12_H_20_NO**

**C_12_H_17_ClNO**

**C_9_H_9_NO**

**C_2_H_2_ClO**


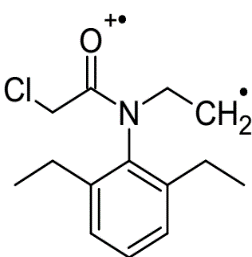

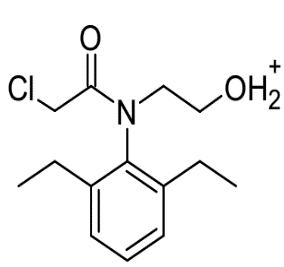
E


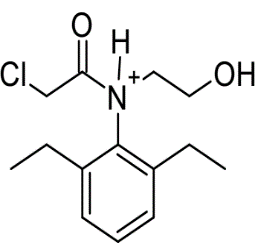

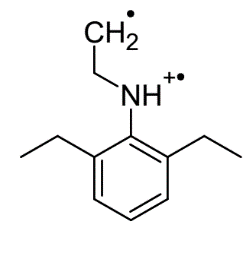
*m/z* 269.9 *m/z* 251.9

*m/z* 269.9 *m/z* 175.9

**Fig. S9** HPLC-MS/MS analysis of the pretilachlor and the intermediate metabolites catalyzed by the strain B2. A, MS2-SCAN mode analysis of the pretilachlor peak (1.1 min); B, Product ion Scan mode analysis of the ion of 312 *m/z*; C, MS2-SCAN mode analysis of the intermediate metabolites peak (0.57 min); D, Product ion Scan mode analysis of the ion of 269.9 *m/z*; E, the proposed structure of characteristic fragment ions from D.
